# Elevated lipoprotein(a) as a predictor for coronary events in older men

**DOI:** 10.1016/j.jlr.2022.100242

**Published:** 2022-06-18

**Authors:** Francesca Bartoli-Leonard, Mandy E. Turner, Jonas Zimmer, Roland Chapurlat, Tan Pham, Masanori Aikawa, Aruna D. Pradhan, Pawel Szulc, Elena Aikawa

**Affiliations:** 1 Division of Cardiovascular Medicine, Department of Medicine, Center for Interdisciplinary Cardiovascular Sciences, Brigham and Women’s Hospital, Harvard Medical School, Boston, MA, USA; 2 INSERM UMR 1033, Hospices Civils de Lyon, University of Lyon, Lyon, France; 3 Division of Cardiovascular Medicine, Department of Medicine, Center for Excellence in Vascular Biology, Brigham and Women’s Hospital, Harvard Medical School, Boston, MA, USA; 4 Division of Preventive Medicine, Brigham and Woman’s Hospital, Harvard Medical School, Boston, MA, USA; 5 Division of Cardiovascular Medicine, VA Boston Medical Centre, Boston, MA, USA

**Keywords:** lipoprotein(a), acute coronary syndrome, CVD, dyslipidemia, atherosclerosis, lipoproteins, vascular biology, ACS, acute coronary syndrome, AMI, acute myocardial infarction, HR, hazard ratio, IHD, ischemic heart disease, Lp(a), lipoprotein (a)

## Abstract

Elevated circulating lipoprotein (a) [Lp(a)] is associated with an increased risk of first and recurrent cardiovascular events; however, the effect of baseline Lp(a) levels on long-term outcomes in an elderly population is not well understood. The current single-center prospective study evaluated the association of Lp(a) levels with incident acute coronary syndrome to identify populations at risk of future events. Lp(a) concentration was assessed in 755 individuals (mean age of 71.9 years) within the community and followed for up to 8 years (median time to event, 4.5 years; interquartile range, 2.5–6.5 years). Participants with clinically relevant high levels of Lp(a) (>50 mg/dl) had an increased absolute incidence rate of ASC of 2.00 (95% CI, 1.0041) over 8 years (*P* = 0.04). Moreover, Kaplan-Meier cumulative event analyses demonstrated the risk of ASC increased when compared with patients with low (<30 mg/dl) and elevated (30–50 mg/dl) levels of Lp(a) over 8 years (Gray’s test; *P* = 0.16). Within analyses adjusted for age and BMI, the hazard ratio was 2.04 (95% CI, 1.0–4.2; *P* = 0.05) in the high versus low Lp(a) groups. Overall, this study adds support for recent guidelines recommending a one-time measurement of Lp(a) levels in cardiovascular risk assessment to identify subpopulations at risk and underscores the potential utility of this marker even among older individuals at a time when potent Lp(a)-lowering agents are undergoing evaluation for clinical use.

Elevated circulating lipoprotein (a) [Lp(a)] is associated with an increased risk of first and recurrent CVD. Circulating levels of Lp(a) are primarily determined via the LPA gene locus (1). Epidemiological (2) and mendelian randomization studies strongly support Lp(a) as a direct cause of coronary artery disease (3), valve stenosis (4), heart failure (5), and peripheral atherosclerosis (6). Recently, both European and Canadian guidelines for the management of dyslipidemias have recognized the utility of measuring Lp(a) within the general population, recommending the assessment of Lp(a) for all individuals at least once in their lifetime, underscoring the importance of Lp(a) on cardiovascular health (7, 8, 9). Conversely, American guidelines recommend that Lp(a) be measured in only select individuals, namely woman with hypercholesterolemia (7). However, conventional lipid panels do not measure Lp(a), and it is still not routinely assessed in the clinic (7), thus highlighting the need for robust evidence toward its impact in CVD. Because of a global aging population, decreasing risk in acute coronary syndrome (ACS) treatment is a priority for clinicians and researchers alike and finding risk factors that may predict outcome is of the highest importance. Currently, percutaneous coronary intervention is commonly used for the treatment of ACS; however, it carries significantly higher risk rates and worse 6-month outcomes in older patients (10, 11). Hence, a better understanding of the contribution of Lp(a) to ACS risk in these at-risk patients is needed to identify which patients may benefit most from Lp(a) therapy. This study set out to investigate elevated Lp(a) levels as a potential predictor of ACS over 8 years in the STRAMBO (STRtructure of the Aging Men’s BOnes) study, a prospective cohort study of 755 community-dwelling older men (aged older than 60 years), who are not on any specific Lp(a)-lowering therapy.

## Materials and methods

### Study cohort

**Fig. 1:**
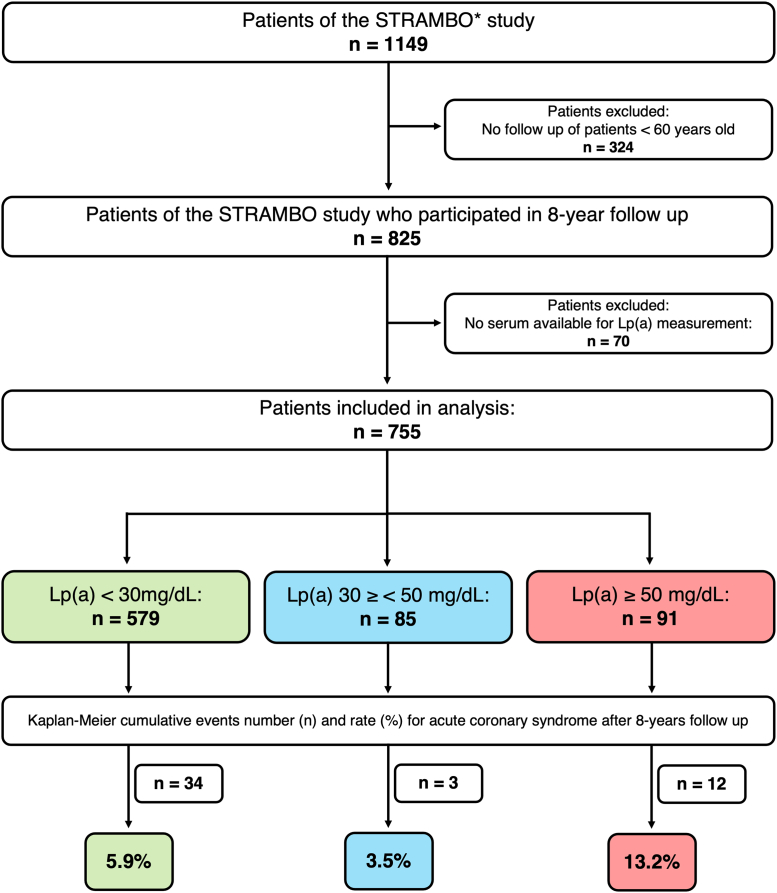
Study design and incidence outcomes. Of the 1,149 male participants enrolled in the STRAMBO study, 755 older than the age of 60 years were assessed for Lp(a) levels at baseline. Kaplan-Meier cumulative event rate for ACS was assessed in three subgroups.

The STRAMBO study is a single-center prospective community-based cohort study on the skeletal fragility and its determinants in men (12, 13). Participants were recruited from 2006 to 2008 from Lyon and surrounding area. All participants were Caucasian. The study was performed as a collaboration between Institut National de la Sante et de la Recherche Medicale and Mutelle de Travailleurs de la Region Lyonnaise France and approved by the local ethics committee in agreement with the Helsinki Declaration. Following informed consent, participants underwent medical exams, with no exclusion criteria applied. Among those included in the original cohort, 825 men older than 60 years were followed up prospectively (14). Of the 755 participants within the study, 112 reported cardiovascular events prior to recruitment, namely acute myocardial infarction (AMI) and ischemic heart disease (IHD). During the baseline visit, all participants completed an interviewer-administered questionnaire, clinical examination, and blood draw. Medications were self-reported and verified via prescriptions. Current smoking status at baseline was self-reported. Pharmacologically treated comorbidities present at baseline (IHD, AMI, and diabetes) were self-reported and dichotomized. Previous incidence of IHD events included interventions such as coronary artery bypass grafting. Blood pressure was measured using a sphygmomanometer following 5 min rest in a reclining position. Medications (statins, fibrates, angiotensin-converting enzyme inhibitors, angiotensin receptor blockers, beta-blockers, diuretics, calcium channel blockers, and vitamin K antagonists) were self-reported and verified using prescriptions before dichotomizing. For the current study, serum was not available for Lp(a) measurement in 70 patients, and thus, the current analysis was performed on 755 participants (Fig. 1).

### End point

All participants received annual questionnaires regarding medical events in the preceding year. When an ACS was self-reported by the participant, the medical records were requested from the corresponding hospital and physician to confirm. The primary end point was ACS defined as a composite of cardiac arrest (reported by proxy), ST-segment-elevation myocardial infarction or non-ST-segment-elevation myocardial infarction, or unstable angina necessitating emergency intervention. Following a participant reaching an end point during follow-up, the participant was not followed up again, with only the first manifestation of ACS was used for analysis. About 34 participants with low, 3 participants with elevated, and 12 participants in with high Lp(a) had an event over the follow-up period.

### Biological assessments

Biological assessments were completed as previously described (13). Nonfasting serum was collected at 13:00 and stored at −80°C with all biological assessments with the exception of Lp(a) and LDL-C conducted within 1 year of collection. LDL-C was determined in 2016 by an Food and Drug Administration-approved homogenous direct method from Roche Diagnostics (Indianapolis, IN) at the Department of Laboratory Medicine, Children’s Hospital Boston, MA as previously described (15). Serum osteoprotegerin was measured by ELISA (Biomedica Medizinprodukte GmbH & Co Kg, Vienna, Austria) as described previously (16). Serum parathyroid hormone was measured using a human-specific two-site immunochemiluminescence assay (ELECSYS; Roche) as described previously (14). Serum 25-hydroxycholecalciferol was measured with RIA (DiaSorin, Stillwater, MN) after acetonitrile extraction (14). High-sensitivity C-reactive protein was measured by immunoturbidimetric latex C-reactive protein assay (17) (Roche Diagnostics, Mannheim, Germany). Serum calcium, phosphorus, and cholesterol were measured using standard laboratory methods. Serum creatinine was measured by the compensated Jaffé’s method. Estimated glomerular filtration rate was calculated using the Chronic Kidney Disease Epidemiology Collaboration equation (18).

Serum Lp(a) was assayed in samples stored at −80°C for >1 year prior to assessment. Lp(a) was assessed using the human Lp(a) sandwich ELISA kit (Abcam; catalog no.: ab212165) according to manufacturer instructions. The ELISA was produced from Lp(a) fragments KIV-2 and KIV-3 taken from isoform P08519, with all standards having the same number of kringle repeats within KIV-2. Sensitivity of the assay was 0.00025 mg/dl with an upper and lower range of 0.00172 and 0.11 mg/dl, respectively. All samples were run in duplicate over 5 days, with the coefficient of variation within the plates 4–11%.

### Statistical analysis

Participants were stratified by Lp(a) into three categories: <30 mg/dl, 30–50 mg/dl, and >50 mg/dl, consistent with prior studies (5, 19, 20, 21). Continuous baseline study characteristics were assessed for normality using the Kolmogorov-Smirnov normality test. Those with Gaussian distribution were assessed via ANOVA, and those with skewed variables were assessed the Kruskal-Wallis test. Categorical variables were compared between groups via Pearson’s Chi-squared test. Absolute risk of ACS was presented as incidence rates per 1,000 person-years. Cumulative incidences between Lp(a) groups were compared using the Gray’s test (22). Kaplan-Meier survival analysis was completed based on Lp(a) cutoffs and ACS events. The risk of ACS was further analyzed using hazard ratios (HRs), estimated via Cox proportional hazards regression. For multifactorial-adjusted Cox proportional hazards regression, potential cofounders were chosen as covariates possibly associated with Lp(a) and ACS, including classical risk factors of age, BMI, smoking, cholesterol, and LDL-C. No missing covariates were present, and thus, none were imputed. All analyses were conducted using RStudio, version 2021.09.0 build 351.

## Results

### Patient population and baseline characteristics

**Fig. 2:**
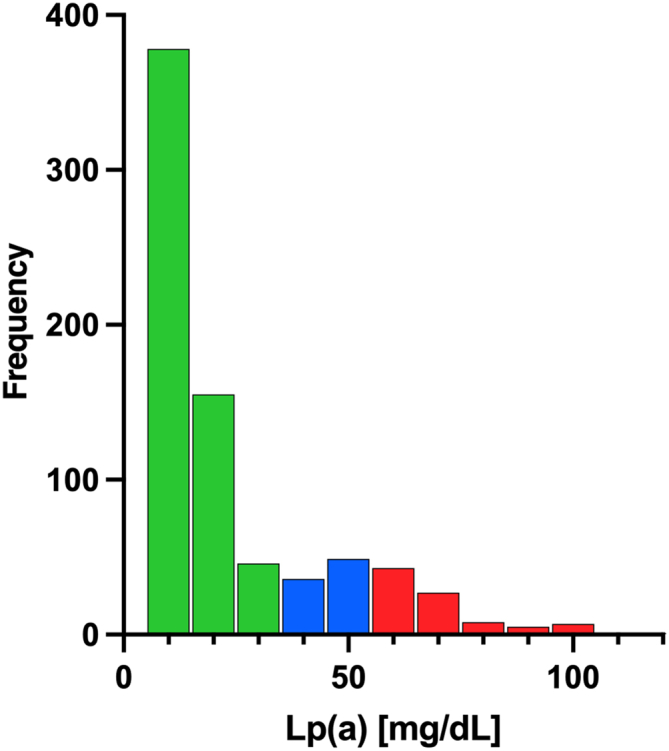
Lp(a) frequency distribution.

Baseline characteristics of the study population are presented in Table 1. Based on clinically relevant cutoffs (5), participants were stratified into three Lp(a) categories: low <30 mg/dl (*n* = 579); elevated 30–50 mg/dl (*n* = 85); and high >50 mg/dl (*n* = 91). The mean age of the study population was 71.5 ± 7.3 years, with no difference in age across the Lp(a) groups (*P* = 0.19). Of the 755 participants within the cohort, 42 reported a previous AMI, with a further 70 being diagnosed with IHD prior to recruitment. BMI, blood pressure, smoking status, and diabetes mellitus status were comparable across the groups. Participants with high Lp(a) were more likely to have a history of IHD (including undergoing a previous coronary artery bypass graft) (*P* = 0.04) but not AMI. Distribution of Lp(a) within participants ranged from 0.2 mg/dl to 120 mg/dl (Fig. 2).

**Table 1:** Baseline characteristics of study population

	Lipoprotein(a) (mg/dl)				
	Total (*n* = 755)	<30 (*n* = 579)	30 ≥ < 50 (*n* = 85)	≥50 (*n* = 91)	*P*
Age	71.9 (65.4–77.6)	72.0 (7.44–77.9)	70.6 (65.8–75.1)	72.1 (65.1–78.4)	0.19
BMI	27.7 (25.2–29.7)	27.6 (25.2–29.9)	27.3 (24.8–29.6)	27.3 (25.0–29.1)	0.20
Weight (kg)	78.6 (71.0–85.0)	78.1 (71.0–85.5)	76.5 (69.0–83.0)	66.9 (70.0–84.0)	0.15
Height (cm)	168.4 (±6.4)	168.3 (±6.4)	167.3 (±5.7)	168.8 (±7.2)	0.47
Systolic blood pressure	134.8 (120.0–140.0)	134.3 (99.2–143.5)	136.9 (120.0–150.0)	134.5 (120.0–147.5)	0.42
Current smoker	47 (6.1%)	34 (5.9%)	2 (2.4%)	8 (8.8%)	0.23
Diabetes mellitus	90 (11.7%)	73 (12.6%)	9 (10.6%)	8 (8.8%)	0.54
History of IHD	115 (14.9%)	82 (14.2%)	9 (10.6%)	21 (23.1%)	0.04
History of AMI	43 (5.6%)	30 (5.2%)	4 (4.7%)	8 (8.8%)	0.35
Statin	201 (26.1%)	138 (23.8%)	26 (30.6%)	29 (31.9%)	0.14
Fibrates	44 (5.7%)	34 (5.9%)	3 (3.5%)	6 (6.6%)	0.62
Angiotensin receptor antagonist	132 (17.1%)	104 (17.9%)	16 (18.8%)	10 (11.0%)	0.24
ACE inhibitor	106 (13.8%)	82 (14.2%)	11 (12.9%)	13 (14.3%)	0.95
Beta-blocker	113 (14.7%)	91 (15.7%)	8 (9.4%)	13 (14.3%)	0.31
Diuretic	185 (24.0%)	145 (25.04)	21 (24.7%)	16 (17.6%)	0.30
Calcium channel blocker	119 (15.5%)	79 (13.6%)	18 (21.2%)	20 (22.0%)	0.04
Vitamin K antagonist	44 (5.7%)	33 (5.7%)	2 (2.4%)	9 (9.9%)	0.10
Cholesterol(mg/dl)	204.5 (±36.9)	199.9 (±37.3)	205.3 (±35.3)	204.2 (±38.9)	0.51
LDL-C (mg/dl)	116.8 (94.4–138.7)	110.5 (93.4–138.2)	123.0 (99.2–143.5)	118.7 (90.7–145.1)	0.10
GFR-CKD-EPI (ml/min)	71.0 (60.7–86.6)	70.8 (60.7–82.6)	73.1 (63.7–84.7)	69.6 (57.4–79.8)	0.35
hsCRP (mg/l)	3.2 (1.6–3.3)	3.0 (0.9–3.3)	2.5 (1.0–2.6)	3.6 (0.8–3.5)	0.80
PTH (pg/ml)	48.2 (33.0–56.0)	48.2 (33.0–55.9)	49.8 (36.5–62.5)	46.5 (30.5–57.0)	0.21
25(OH)D (ng/ml)	22.0 (15.0–28.0)	21.9 (15.0–28.0)	21.7 (16.0–26.0)	23.2 (15.4–29.8)	0.70
OPG (pmol/l)	4.1 (3.2–4.8)	4.2 (3.2–4.9)	3.8 (3.0–4.5)	4.1 (3.0–4.0)	0.26
Ca (mmol/l)	2.4 (2.3–2.4)	2.4 (2.3–2.4)	2.3 (2.3–2.5)	2.3 (2.3–2.4)	0.44
P (mmol/l)	1.0 (±0.2)	1.0 (±0.2)	1.0 (±0.2)	1.1 (±0.2)	0.27

BP, blood pressure; Ca, calcium; GFR CKD-EPI, glomerular filtration rate Chronic Kidney Disease Epidemiology Collaboration; hsCRP, high-sensitivity C-reactive protein; OPG, osteoprotegerin; P, phosphorus; PTH, parathyroid hormone.

Data are presented as mean ± SD, *n* (%), or median (interquartile range).

### Absolute risk of ACS is associated with elevated Lp(a) levels

**Fig. 3:**
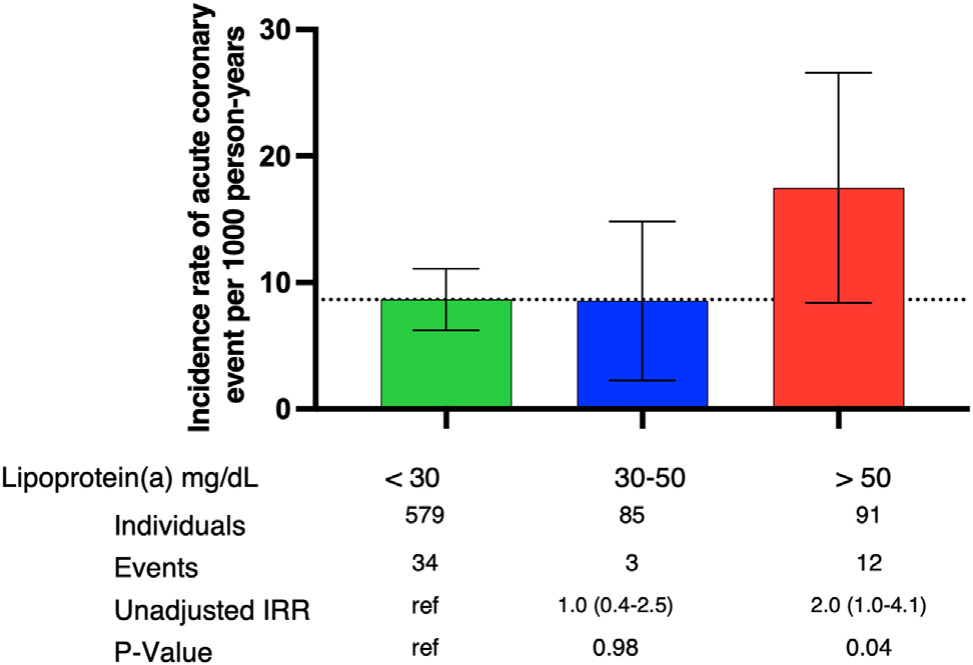
Absolute risk of ACS stratified via circulating Lp(a) levels. Data based on 755 individuals from the STRAMBO cohort. Incidence rates and incidence rate ratios (IRRs) with 95% confidence intervals.

Median follow-up time to event was 4.5 years (interquartile range, 2.5–6.5 years), during which 49 individuals had an ACS event. About 16% of participants without an event did not complete the 8-year follow-up. The incident rate (number of events per 1,000 person-years at risk) of ACS was greatest in those with high (>50 mg/dl) levels of Lp(a) (*P* = 0.04); however, there was no difference between low (<30 mg/dl) and elevated Lp(a) (30–50 mg/dl) levels (Fig. 3). Individuals with elevated Lp(a) levels had an incidence rate of 8 per 1,000 person-years for ACS, whilst the incidence rate for those with high (>50 mg/dl) Lp(a) levels was 17. In participants with Lp(a) greater than 30 mg/dl at baseline, total incidence of ACS was 8.5% of the population. Within the unadjusted absolute incidence rate ratio with low (<30 mg/dl) Lp(a) as a reference, the incidence rate ratio was 1.0 (0.4–2.5) for individuals with elevated (30–50 mg/dl) Lp(a) and 2.0 (1.0–4.1) for those with high (>50 mg/dl) Lp(a) levels.

**Supplemental Fig. S1:**
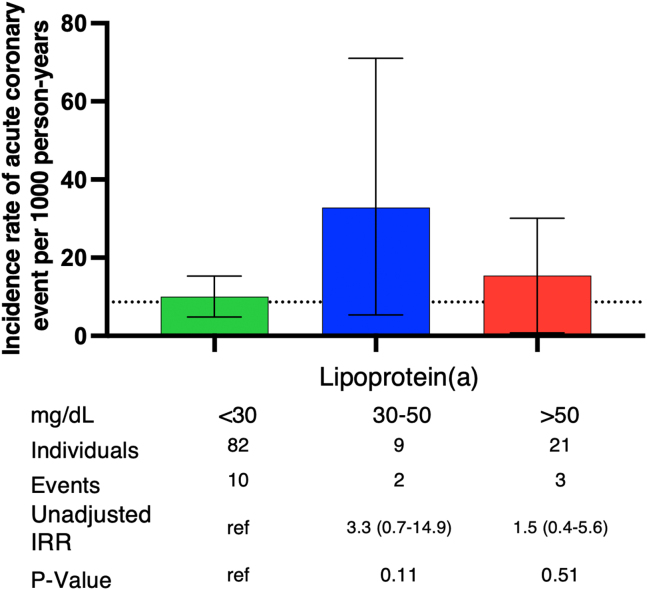
**Absolute risk of acute coronary syndrome in participants with previous acute myocardial infarction or ischemic heart disease according to concentrations of lipoprotein(a).** Data based on 112 individuals from the STRAMBO cohort. Incidence rates and incidence rate ratios with 95% confidence intervals. IRR indicates incidence rate ratio

**Supplemental Fig. S2:**
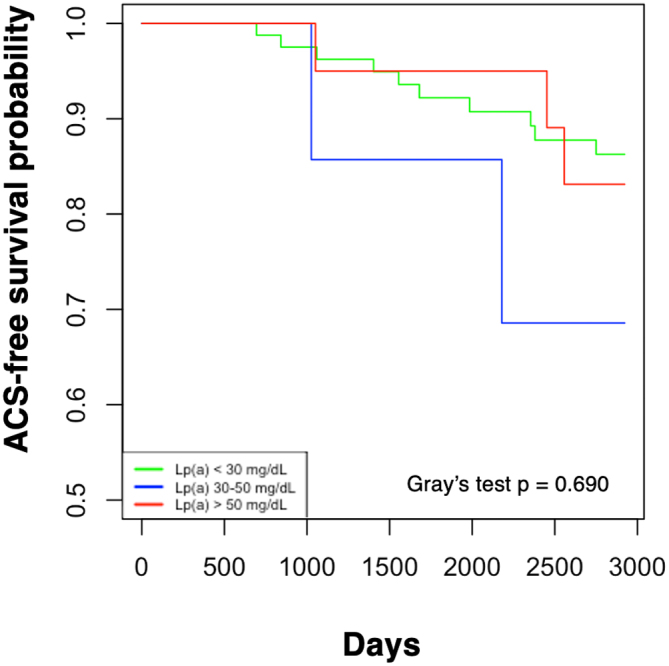
**Kaplan-Meier curve in participants with previous acute myocardial infarction or ischemic heart disease corresponding with low, elevated and high lipoprotein(a) levels for 8-year follow up of acute coronary syndrome.** Low Lp(a) corresponds with a value under 30 mg/dl, elevated Lp(a) corresponds with a value between 30 and 50 mg/dl and high Lp(a) is levels over 50 mg/dl

Within a subanalysis of participants with prior AMI or IHD diagnosis (*n* = 112), absolute incidence rate of ACS was 1.54 (0.4–5.6) in the high (>50 mg/dl) Lp(a) group and further increased to 3.3 (0.7–14.9) in the elevated (30–50 mg/dl) Lp(a) group when compared with low (<30 mg/dl) reference group (supplemental Table S1). Of the 112 participants with elevated or high levels of Lp(a), 4.5% of the participants had an ACS event within the 8-year follow-up (supplemental Fig. S1). Notably, the elevated Lp(a) subpopulation had a greater incidence rate than the high Lp(a) cohort, which may be in part, because of the high levels of Lp(a) leading to early death and confounding this aspect of analysis; however, this is not confirmed. Comparatively, in a subanalysis of participants without prior AMI or IHD diagnosis (*n* = 558), absolute incidence ratio was 1.7 (0.4–7.4) in the elevated Lp(a) when compared with low (<30 mg/dl) reference group (*P* = 0.45) (supplemental Table S2). Within the high Lp(a) group, absolute risk ratio was significantly higher at 3.2 (1.4–7.6, *P* = 0.004) when compared with control, with the incidence rate of 27 per 1,000 person-years. Notably, in participants without previous AMI or IHD, Lp(a) shows a positive correlation with absolute risk compared with those with previous disease, in which Lp(a) over 30 mg/dl appears to increase incidence risk. However, neither subanalyses showed a significant equality of cause-specific cumulative incidence risk (supplemental Figs. S2 and S4).

### Elevated Lp(a) levels are associated with ACS in 8-year follow-up

**Fig. 4:**
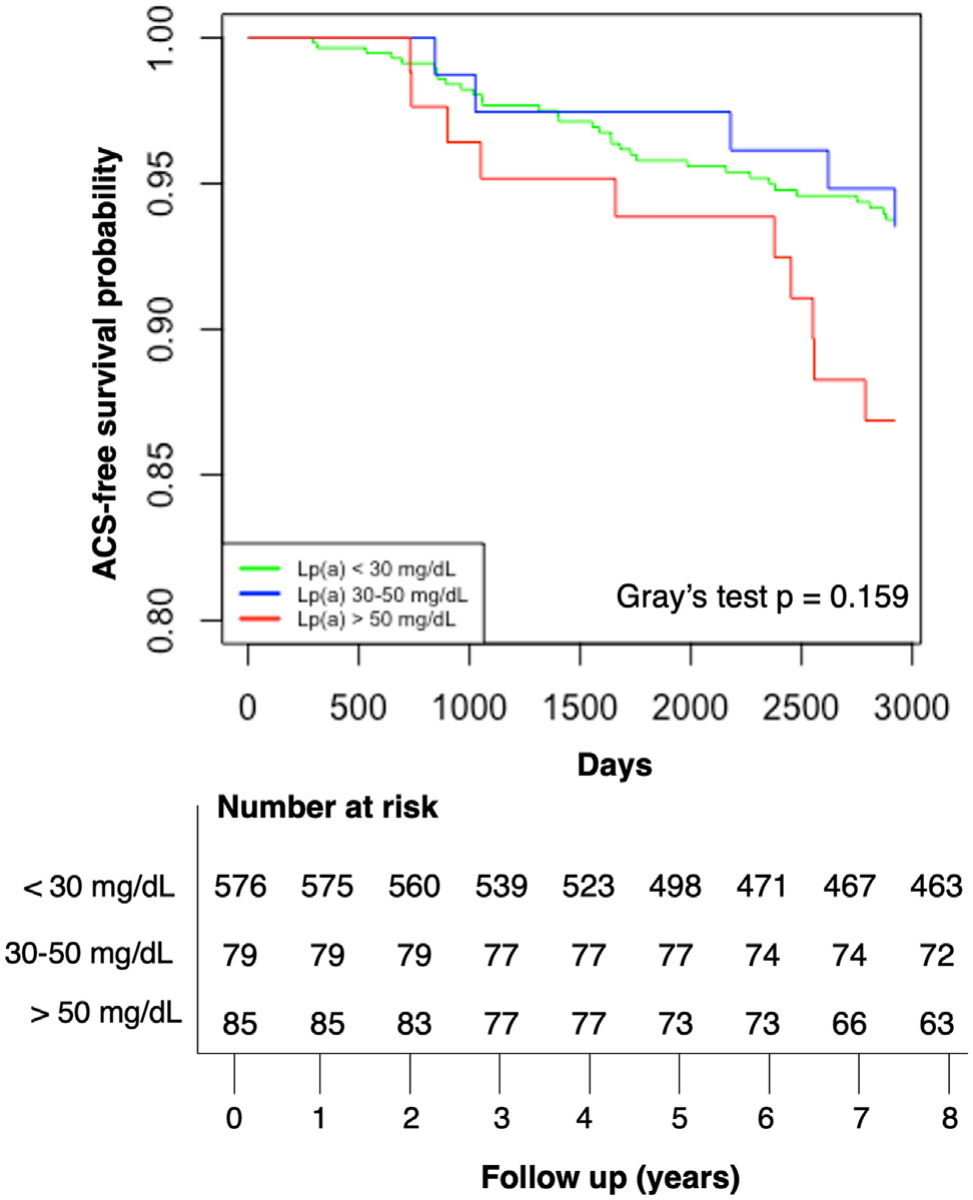
Kaplan-Meier curve corresponding with low, elevated, and high Lp(a) levels for 8-year follow-up of ACS. Low Lp(a) corresponds with a value under 30 mg/dl, elevated Lp(a) corresponds with a value between 30 and 50 mg/dl, and high Lp(a) is with levels over 50 mg/dl.

**Fig. 5:**
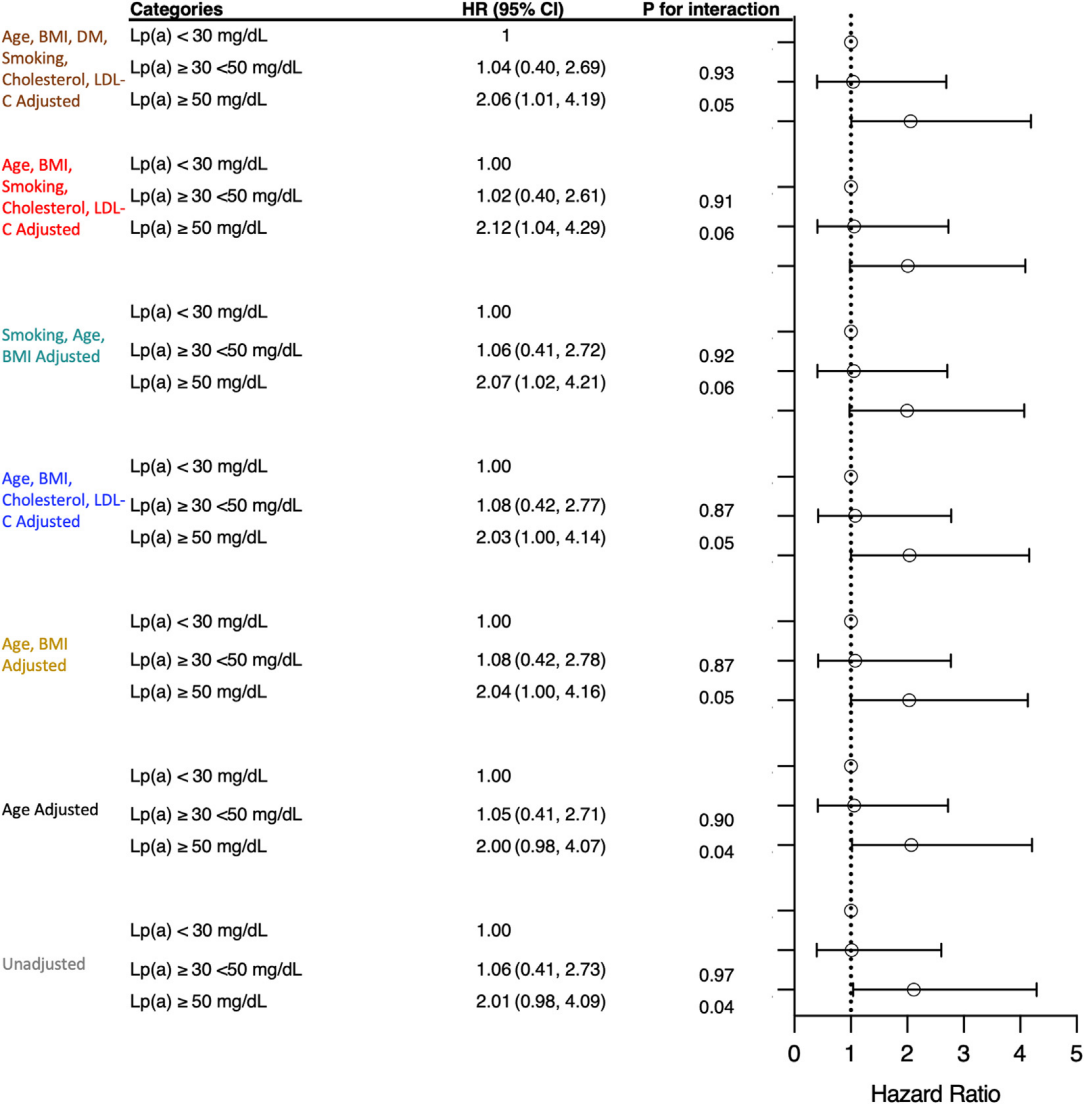
Cox regression analysis corresponding with low, elevated, and high Lp(a) levels for 8-year follow-up of ACS. Low Lp(a) levels <30 mg/dl, elevated Lp(a) levels <30–50 mg/dl, and high Lp(a) levels >50 mg/dl. Unadjusted HRs (white), adjusted HRs for age (black), adjusted HRs for age and BMI (yellow), adjusted HRs for age, BMI, cholesterol, LDL-C (blue), adjusted HRs for smoking, age, BMI (green), adjusted HRs for smoking, age, BMI cholesterol, LDL-C (red), and adjusted HRs for smoking, age, BMI cholesterol, LDL-C, and diabetes mellitus (brown).

Kaplan-Meier cumulative event rate for ACS after 8-year follow-up was 5.9% in the low Lp(a) group (<30 mg/dl), 5.8% in the elevated (30–50 mg/dl) Lp(a) group, and 10.9% in the high (>50 mg/dl) Lp(a) group (Fig. 4). Within a Cox regression model, HRs were higher in those with high (>50 mg/dl) Lp(a) levels with and without the adjustment for several covariates. Univariate analysis HRs were 1.1 (0.4–2.7) in elevated Lp(a) (30–50 mg/dl) and 2.0 (1.0–4.1) (*P* = 0.04) in the high (>50 mg/dl) Lp(a) group, with low Lp(a) (<30 mg/dl) as the reference, which remained significant after the adjustment of age, BMI, cholesterol, and LDL-C sequentially (Fig. 5).

In analyses stratified by Lp(a) level, adjusted for age and BMI, HRs were 1.1 (0.4–2.8) in the elevated (30–50 mg/dl) Lp(a) group and 2.0 (1.0–4.1) (*P* = 0.04) in the high (>50 mg/dl) Lp(a) group. When adjusting for age, BMI, cholesterol, and LDL-C HRs were 1.1 (0.4–2.8) in the elevated (30–50 mg/dl) Lp(a) group and 2.1 (1.0–4.2) (*P* = 0.05) in the high Lp(a) group. Adjusting for smoking, age and BMI HRs were 1.1 (0.4–2.7) in elevated (30–50 mg/dl) Lp(a) group and 2.1 (1.0–4.2) (*P* = 0.06) in high (>50 mg/dl) Lp(a) group.

When adjusting for age, BMI, smoking, cholesterol, and LDL-C HRs were 1.0 (0.4–2.6) in the elevated (30–50 mg/dl) Lp(a) group and 2.1 (1.0–4.3) (*P* = 0.06) in high Lp(a) group. Finally, when adjusting for all covariates, age, BMI, diabetes mellitus, smoking, cholesterol, and LDL-C, HRs were 1.0 (0.4–2.7) in the elevated (30–50 mg/dl) Lp(a) group and 2.1 (1.0–4.2) (*P* = 0.05) in high Lp(a) group, thus demonstrating a limited impact on other traditional risk factors on the impact of Lp(a) in ACS development.

## Discussion

This single-center prospective cohort study investigated the association of Lp(a) levels with ACS events in older community-dwelling men (median follow-up time = 4.5 years). Serum levels of Lp(a) greater than 50 mg/dl associated with increased risk of ACS after adjustment for multiple covariates, including LDL-C, cholesterol, age, and BMI. No difference, however, was observed between elevated (30–50 mg/dl) and low levels (<30 mg/dl) of Lp(a), either in absolute 8-year risk or hazard regression analysis. High levels of Lp(a) at baseline did not correlate with any classical risk factors of ACS; age, high blood pressure, cholesterol, smoking, or diabetes. The lack of synergistic effect between Lp(a) and cholesterol (*r* = 0.054) and LDL-C (*r* = 0.082) further suggests the independent role Lp(a) provides in prospectively predicting future ACS events; however, both these correlations require a much greater sample size; 3,874 and 1,165, respectively, to be appropriately powered. Current Canadian and European guidelines for dyslipidemia management suggest a one-time measurement of Lp(a) for all patients, especially for those at high risk of CVD (20, 23). Recently, two large placebo-controlled trials of cholesterol-lowering proprotein convertase subtilisin kexin 9 inhibitors have demonstrated a secondary effect of reducing circulating Lp(a) (24). Reductions of Lp(a) by alirocumab were associated with a reduction in primary peripheral arterial events, venous thromboembolism, and major cardiovascular events, independent of LDL-C reduction, supporting the concept that circulating Lp(a) is a modifiable CVD risk factor.

Recent advances of RNA technology have led to the development of Lp(a)-specific oligonucleotide-based therapies, such as mipomersen, which target ApoB (25); and pelacarsen, which targets Apo(a) (26). Mipomersen however is more potent for LDL than Lp(a), demonstrating best results in hypercholesterolemia patients (27), whereas the antisense oligonucleotide, pelacarsen, directly targets the cholesterol portion of Lp(a), Lp(a)-C, reducing levels up to 80% (28). These agents operate independently of isoform size and genetic variant and have shown promising results in clinical trials, with further therapies such as Amgen’s RNAi ARC-LPa, targeting of LPA mRNA in early development (29). More recently, siRNA targeting LPA mRNA was trialed in a small study (*n* = 32). A single dose of siRNA SLN360 was given to increase its selective uptake and concentration within hepatocytes, binding and degrading the apo(a) mRNA and reducing overall Lp(a) plasma levels for 150 days (30). With studies such as these highlighting the urgent need for Lp(a)-specific therapies, the current guidelines must still focus on managing and mitigating the risk associated with high Lp(a) levels prior to the need for intervention until a specific therapy is widely available (7, 8, 31). Within the current study, these data demonstrate that integration of classical risk factors into HR models does not alter the risk for ACS, further evidencing the critical importance of incorporating Lp(a) levels in patient treatment plans. These findings support the latest guidelines from both the European and Canadian guiding bodies, which recommend a one-time Lp(a) screening for all, irrespective of CVD risk and other risk factors (7, 8, 31).

**Supplemental Fig. S3:**
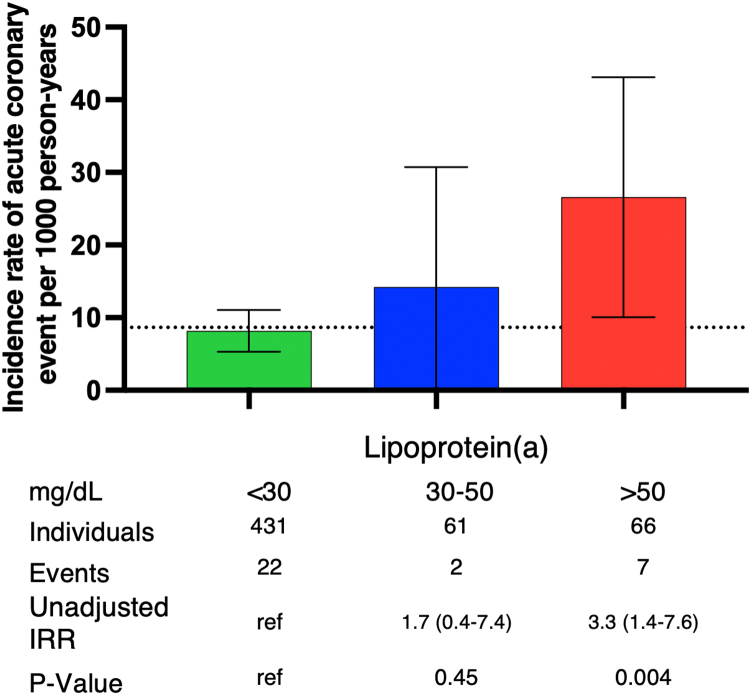
**Absolute risk of acute coronary syndrome in participants without previous acute myocardial infarction or ischemic heart disease according to concentrations of lipoprotein(a).** Data based on 557 individuals from the STRAMBO cohort. Incidence rates and incidence rate ratios with 95% confidence intervals. IRR indicates incidence rate ratio

**Supplemental Fig. S4:**
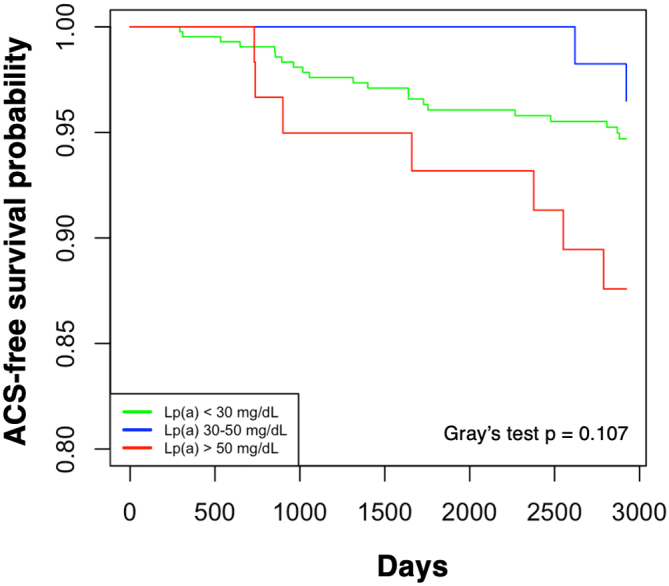
**Kaplan-Meier curve in participants without previous acute myocardial infarction or ischemic heart disease corresponding with low, elevated and high lipoprotein(a) levels for 8-year follow up of acute coronary syndrome.** Low Lp(a) corresponds with a value under 30 mg/dl, elevated Lp(a) corresponds with a value between 30 and 50 mg/dl and high Lp(a) is levels over 50 mg/dl

Despite strong clinical evidence that reductions in Lp(a) could meaningfully reduce CVD risk (32), no specific and effective therapeutic agents targeting Lp(a) are currently available (33). Recent studies have demonstrated that Lp(a) remains a risk factor in individuals who are currently undergoing treatment with statins (34) and in those with prior cardiovascular events on secondary prevention therapies (35). Statins have been shown to modestly increase Lp(a) when compared with placebo (36), thus demonstrating the urgent need for targeted Lp(a) treatment. Despite the increase in Lp(a) levels, statins remain at the frontline of treatment for those with high cholesterol. Evidence is available from mechanistic, observational, and Mendelian randomization studies to support a causal role of Lp(a) in the development of a multitude of CVD subtypes (32, 37, 38, 39), within both a primary (40, 41) and secondary (35) setting, but few studies aside from The Copenhagen City Heart Study (42, 43) have undertaken such evaluations over such a long period. While underpowered, these data may suggest that elevated Lp(a) could play a more important role in those patients who have already undergone a previous AMI or IHD (supplemental Figs. S1 and S2), in comparison to those with no previous CVD (supplemental Figs. S3 and S4). However, these finding would need to be assessed in a larger cohort to confirm. Notably, within the whole population, when adjusting for covariates in a Cox hazard proportional model, Lp(a) is significantly influenced by LDL-C in those patients with previous CVD. Conversely, LDL-C has no significant impact in participants with previous AMI and IHD, despite no significant difference in LDL-C levels within the groups in both the total population assessed and the prior CVD cohort.

Utilizing absolute cutoffs of Lp(a), this study identified an increased risk of ACS in those with Lp(a) values >50 mg/dl. Previous studies with quintile or quartile stratification of Lp(a) analysis have identified this increased risk (3); however, the application of absolute cutoff values of Lp(a) is useful to support future clinical integration of this measurement. Whilst absolute cutoffs may be beneficial for decision making within the clinic, a linear increase in atherosclerotic CVD risk has been observed with Lp(a) concentration, suggesting that even a modest increase in Lp(a) may be determinantal (23). Notably, within this study, the observation that no difference was found between low (<30 mg/dl) and elevated (30–50 mg/dl) Lp(a) in ACS risk differs from recent findings, where Lp(a) levels over 30 mg/dl contributed to an increased risk of coronary heart disease (44) and a linear relationship with both fatal and nonfatal cardiovascular risk (45, 46). This may, in part, be due to the disparity in race between the two studies. The aforementioned study focused on a Chinese cohort. Chinese populations are demonstrated to have lower Lp(a) levels than Caucasians (47), and thus an elevation of Lp(a) of over 30 mg/dl in a Chinese population may be comparable to a 50 mg/dl or greater Lp(a) level in Caucasians, therefore providing a potential explanation for the discrepancy in risk.

Moreover, within this study, the absolute risk of developing ACS with 8 years of baseline more than doubled within participants with Lp(a) over 50 mg/dl. This risk is greater than seen in previous studies that have integrated absolute cutoffs of Lp(a) and CVD risk. Previous observations have demonstrated the risk of Lp(a) in short-term follow-up studies (predominantly 5 years or less) and in a secondary prevention setting, but only a few reports have assessed Lp(a) as a long-term predictive measure in mixed primary and secondary prevention community-based cohorts in an older than 60 population (48). A meta-analysis of placebo-controlled randomized statin trials, which combined both primary and secondary prevention populations, demonstrated that individuals with high Lp(a) levels (>50 mg/dl) had an age- and sex-adjusted HR of 1.31 in CVD, compared with those with Lp(a) levels under 15 mg/dl. Similarly, in a large observational Danish prospective cohort study, those with Lp(a) values between 50 and 100 mg/dl had an HR of ∼1.5 compared with those with Lp(a) <10 mg/dl. Thus, the findings presented here provide further weight to the rationale for clinical measures of Lp(a) to be taken routinely and prior to development of a CVD and highlighting its value as a predictive measure.

Strengths of this analysis involve the length of follow-up. In the current study, the population had an average age of 71.5; however, subsequent ACS occurred on average of 5 years later. Therefore, this report demonstrates the importance of studies with long follow-up periods, as current short-term studies may miss the true impact of Lp(a) on ACS outcomes. In addition, this study used the same commercially available standardized assay with minimal isoform bias measured over a short period (5 days) for all samples, facilitating low interassay variability. Measurement of Lp(a) is currently not uniformly calibrated and standardized, and studies which utilize different methods over long periods will result in variable absolute measurements. Conversely, this study is not without limitations. The present investigation is limited in that only Caucasian males residing in France were recruited in the study population. However, these results are consistent with reports that included more diverse ethnic backgrounds and those assigned female at birth. There are currently conflicting results in the literature regarding sex differences in the predictive value of Lp(a), showing both no association of Lp(a) in large prospective cohorts in a female-only subset analysis (48) and also contrary findings that females have a stronger relative risk with regard to Lp(a) than men in a secondary prevention setting (49, 50, 51). Second, nonfasting blood draws were used for Lp(a) measurement, which could influence values; however, the use of nonfasting lipid profiles is now widely endorsed in risk assessment.

## Conclusions

The current single-center prospective study of Lp(a) levels and ACS association adds support for recent guidelines recommending a one-time measurement of Lp(a) in cardiovascular risk assessments to identify subpopulations at risk with markedly elevated levels of Lp(a) sufficient to independently contribute to ACS risk. This work further provides support for the ongoing programs to develop efficacious Lp(a)-lowering drugs and understanding of the mechanisms behind Lp(a)-associated CVD. Overall, this study provides a rationale for the dire need to develop Lp(a)-lowering drugs, because of their significance on ACS development, irrespective of other CVD risk factors.

## Data availability

Data are available upon request from Elena Aikawa e.aikawa@bwh.harvard.edu.

## Supplemental data

This article contains supplemental data.

## Conflict of interest

The authors declare that they have no conflicts of interest with the contents of this article.

## Figures and Tables

**Supplemental Table S1:**
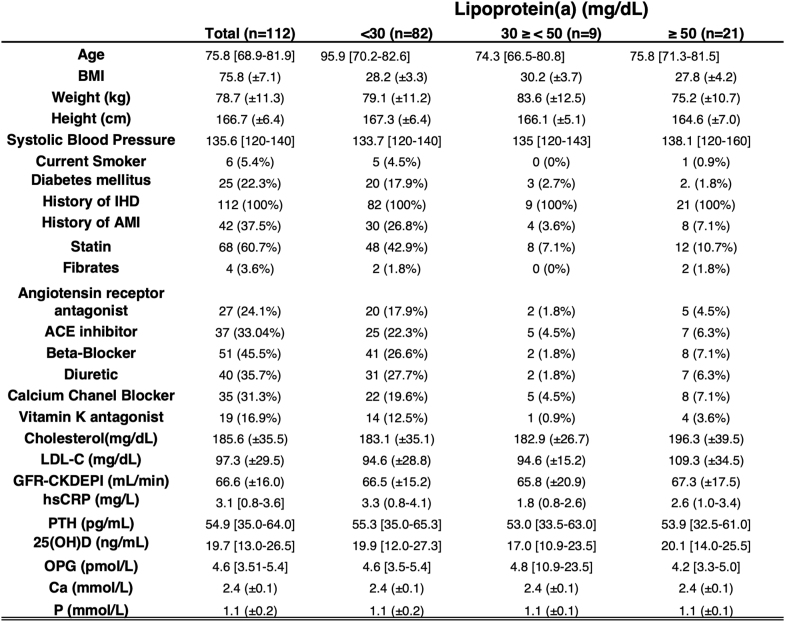
Baseline characteristics of population with previous acute myocardial infarction or ischemic heart disease. Data presented as mean ± SD, n (%), or median (IQR). ACEi angiotensin-converting-enzyme inhibitor; AMI acute myocardial infarction; BMI indicates body mass index; BP, blood pressure; Ca, calcium; CKD-EPI GFR, Glomerular filtration rate-chronic kidney disease epidemiology collaboration; hsCRP high sensitivity c-reactive protein; IHD Ischemic heart disease; LDL-C low density lipoprotein-C; OPG osteoprotegerin; P, phosphate; PTH parathyroid hormone.

**Supplemental Table S2:**
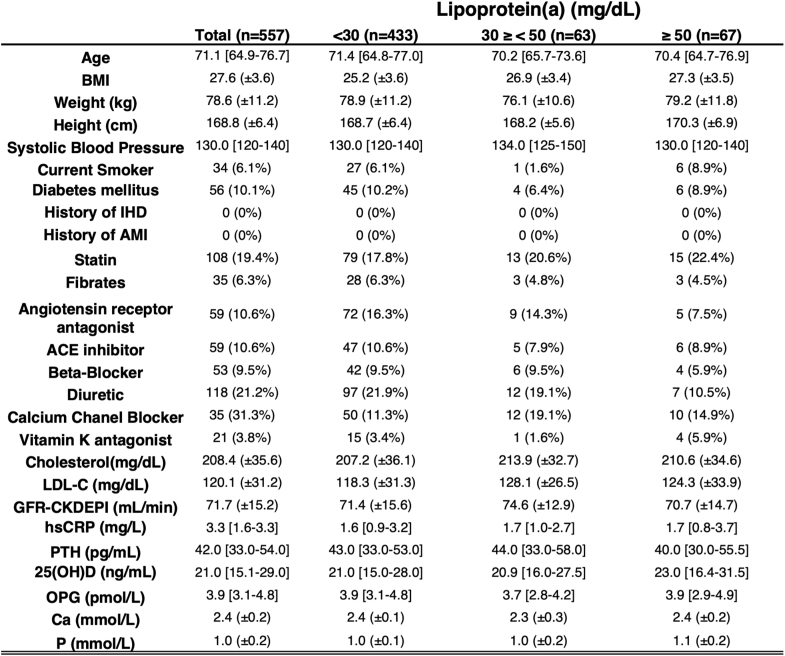
Baseline characteristics of population without previous acute myocardial infarction or ischemic heart disease. Data presented as mean ± SD, n (%), or median (IQR). ACEi angiotensin-converting-enzyme inhibitor; AMI acute myocardial infarction; BMI indicates body mass index; BP, blood pressure; Ca, calcium; CKD-EPI GFR, Glomerular filtration rate-chronic kidney disease epidemiology collaboration; hsCRP high sensitivity c-reactive protein; IHD Ischemic heart disease; LDL-C low density lipoprotein-C; OPG osteoprotegerin; P, phosphate; PTH parathyroid hormone.
